# P2X_7_ accelerate tissue fibrosis via metalloproteinase 8‐dependent macrophage infiltration in a murine model of unilateral ureteral obstruction

**DOI:** 10.14814/phy2.15878

**Published:** 2023-11-23

**Authors:** Jacob Rudjord Therkildsen, Stine Julie Tingskov, Michael Schou Jensen, Helle Praetorius, Rikke Nørregaard

**Affiliations:** ^1^ Department of Biomedicine Aarhus University Aarhus C Denmark; ^2^ Department of Clinical Medicine Aarhus University Aarhus C Denmark; ^3^ Department of Clinical Biochemistry Aarhus University Hospital Aarhus N Denmark

**Keywords:** antifibrotic, gender difference, macrophage, Metalloprotease 8, P2X_7_, renal fibrosis

## Abstract

Renal fibrosis is tightly associated with chronic kidney disease, irrespective of the underlying pathogenesis. We previously demonstrated mild antifibrotic effects of targeting the P2X_7_ receptor in a pyelonephritis model. Reduced P2X_7_R‐activation elevated the neutrophil‐to‐macrophage ratio, resulting in less matrix accumulation without affecting the initial tissue healing. Here, we test if this P2X_7_R‐dependent modification of matrix accumulation also applies to a noninfectious fibrosis model of unilateral ureteral obstruction (7dUUO) and whether the response is gender‐dependent. We found that P2X_7_^−/−^ mice show reduced fibrosis compared to wild type after 7dUUO: the effect was most pronounced in females, with a 55% decrease in collagen deposition after 7dUUO (*p* < 0.0068). P2X_7_R deficiency did not affect early fibrosis markers (TGF‐β, α‐SMA) or the renal infiltration of neutrophils. However, a UUO‐induced increase in macrophages was observed in wildtypes only (*p* < 0.001), leaving the P2X_7_^−/−^ mice with ≈50% fewer CD68^+^ cells in the renal cortex (*p* = 0.018). In males, 7dUUO triggered an increase in diffusely interstitial scattering of the profibrotic, macrophage‐attracting metalloproteinase MMP8 and showed significantly lower MMP8 tissue expression in both male and female P2X_7_^−/−^ mice (*p* < 0.0008). Thus, the P2X_7_R is advocated as a late‐stage fibrosis moderator by reducing neutrophil‐dependent interstitial MMP8 release, resulting in less macrophage infiltration and reduced matrix accumulation.

## 1 INTRODUCTION

Renal fibrosis is an essential component of chronic kidney disease (CKD) regardless of the etiology of renal insufficiency. The condition constitutes a substantial part of the overall societal and medical challenges, with a global prevalence of 10%–15% (Hill et al., 2016). Compared to other epithelial organs, such as the intestines or the liver, the kidneys have a relatively low capacity for regeneration. As a result, the renal function deteriorates with advancing age, reflecting graduate loss of nephrons over time, presumably because of cumulative single damaging events to the nephrons. Renal fibrosis is known to coincide with the progression of CKD, whether it originates from glomerular damage or tubular injury (Eitner & Floege, 2003), and, at present, renal fibrosis is the best predictor of progression of renal failure‐associated CKD (Majo et al., 2019). Therefore, modulation of fibrosis has been a hallmark in the strategy toward slowing the progression of renal insufficiency characterizing chronic kidney failure. However, an important question is whether fibrosis as a process accelerates nephron loss or is merely a by‐product of the underlying renal damage mainly responsible for the deterioration of renal function.

Several preclinical studies have identified potential targets for intervening with or even reversing renal fibrosis. Unfortunately, none of these strategies has been confirmed to provide reno‐protection in clinical trials (for review, see Ruiz‐Ortega et al., 2020). In this review, it was also pointed out that the exploited targets mainly influence the early phases of fibrosis during the inflammatory reaction to the primary insult (Ruiz‐Ortega et al., 2020). Since fibrosis is an essential component of normal tissue healing upon acute damage, counteracting the early healing processes might have the opposite of the intended effect on tissue function.

Thus, we speculate that renal fibrosis disturbs renal function not primarily by compromising the function of neighboring segments but by increasing the diffusion distances between the renal tubules and vasculature, which is a prerequisite for normal glomerular and tubular function. Therefore, we propose a strategy for intervening in the delicate balance between matrix production and resorption, limiting the overall quantity of extracellular matrix in response to injury. We have previously shown that either a genetical or pharmacological reduction of P2X_7_ receptor (P2X_7_R) function dampens the late stages of fibrosis in response to pyelonephritis by pushing the balance between neutrophil and macrophage infiltration (Therkildsen et al., 2019). The P2X_7_R has notoriously been shown to be implicated in acute inflammation. The natural ligand for the P2X_7_R is extracellular ATP, a paracrine factor belonging to the group of pathogen and damage‐associated molecular pattern (PAMP and DAMP) molecules with a primarily pro‐inflammatory profile. ATP has been shown to promote chemotaxis in neutrophils and macrophages to the wounded or infectious sites in a process that involves several P2‐receptor subtypes (Elliott et al., 2009). Extracellular ATP has also been shown to be profibrotic (Belete et al., 2011; Haanes et al., 2012; Huang et al., 2014; Kunzli et al., 2007; Mezzaroma et al., 2011; Moncao‐Ribeiro et al., 2014; Riteau et al., 2010) by promoting the release of interleukin 13 (IL‐13) and transforming growth factor β (TGF‐β) from lymphocytes and macrophages with downstream activation of local fibroblasts (Elliott et al., 2009; Maitre et al., 2015). Moreover, P2X_7_R has notoriously been implicated in the Nod‐like receptor protein 3 (NLRP3) inflammasome–caspase 1 dependent cleavage of pro‐IL‐1β and release of IL‐1β (Kahlenberg & Dubyak, 2004; Qu et al., 2007).

Here, we confirm that lack of P2X_7_R expression dampens renal fibrosis, also in response to UUO, an effect that is more extensive in female mice. We found that despite the P2X_7_R having a profound impact on acute inflammation, the acute renal damage and the early fibrotic markers were unchanged in the P2X_7_^−/−^ compared to the wild type. However, the lack of P2X_7_ receptor function markedly reduces macrophage infiltration, which increases the neutrophil‐to‐macrophage ratio. Interestingly, we find that the release and tissue distribution of MMP8 is substantially reduced in the P2X_7_ receptor‐deficient mice, and we suggest that preventing P2X_7_ receptor activation on neutrophils prevents the release of MMP8, thus diminishing macrophage infiltration and macrophage‐dependent formation of extracellular matrix.

## 2 MATERIALS AND METHODS

### 2.1 Animals

P2X_7_^−/−^ mice backbred over 15 generations into a BALB/cJ background were kept and bred at the Institute of Biomedicine, Aarhus University (Aarhus, Denmark), with the routine of reintroducing BALB/cJ at a frequency adapted to the production rate. The P2X_7_^−/−^ mice were matched with either P2X7^+/+^ littermates from heterozygous breeding or age‐matched BALB/cJ mice from Janvier Labs (Saint‐Berthevin, France). The P2X_7_^−/−^ mice were originally developed by GlaxoSmithKline (London, UK). Animal experiments were conducted in line with the Federation of European Laboratory Animal Science Associations guidelines and Danish national legislation. Permission to conduct these experiments was obtained from the Danish Animal Experiments Inspectorate (Dyreforsøgstilsynet, Glostrup, Denmark; 2015‐15‐0201‐00658).

### 2.2 A murine model of unilateral ureteral obstruction

Experiments were performed in female and male BALB/cJ mice of 7–8 weeks. For weight and other characteristics, please see Table 1. Animals had access to standard rodent chow (Altromin, Lage, Germany) and tap water ad libitum. Animals were allowed to acclimatize for 7 days in the animal facility at the Department of Clinical Medicine, Aarhus University, before surgery. Male and female mice were allocated into the following experimental groups: Sham‐operated (1) wild type and (2) P2X_7_^−/−^ mice and 7 days of UUO (7dUUO) induced in (3) wild‐type mice and (4) P2X^7−/−^ mice. For all groups, six males and six females were included, and the operations were carried out in a blinded fashion. On the day of surgery, mice were anesthetized with 2% sevoflurane (Abbott Scandinavia AB, Solna, Sweden) mixed with atmospheric air at 2 L/min and injected with buprenorphine (Temgesic, Indivior UK Limited, Berkshire, UK). After an abdominal midline incision, the left ureter was located and occluded with a 6–0 silk ligature. Sham‐operated animals were subjected to the same procedure except for ligation. Additionally, buprenorphine was administered if mice showed signs of distress after either operation. After 7 days of UUO, the mice were terminated, and the obstructed or sham‐operated kidney was divided into one half for submission fixation and one half divided into preparations for mRNA and protein isolation (stored at −80°C). Plasma samples were kept at −80°C until further processing.

**TABLE 1:** Body weight, kidney weight, and fluid homeostasis values.

	Sham WT	Sham KO	7dUUO WT	7dUUO KO	Sham WT	Sham KO	7dUUO WT	7dUUO KO
Body weight (g)	19.3 ± 1.4	20.2 ± 1.3	18.8 ± 1.6	20.6 ± 2.1	23.5 ± 1.9	27.9 ± 2.4*	21.9 ± 9.1	22.6 ± 1.4**
Left kidney weight (g/25 g BW)	0.14 ± 0.06	0.16 ± 0.01	0.22 ± 0.02*	0.21 ± 0.02**	0.19 ± 0.02	0.20 ± 0.01	0.26 ± 0.03*	0.23 ± 0.02
Plasma osmolality (mOsmol/kg)	335.6 ± 9.1	341.1 ± 20.2	331.8 ± 7.0	338.0 ± 10.5	332.4 ± 6.3	339.0 ± 4.3	336.4 ± 8.2	342.4 ± 15.8
Plasma Sodium (mmol/L)	150.6 ± 1.1	150.7 ± 1.9	147.7 ± 1.2	151.7 ± 1.1***	151.0 ± 0.1	152.5 ± 0.8	150.8 ± 1.5	153.4 ± 3.6
Plasma Potassium (mmol/L)	4.8 ± 0.3	4.7 ± 0.5	4.6 ± 0.4	4.0 ± 0.2	5.0 ± 0.6	4.5 ± 0.3	4.6 ± 0.3	4.3 ± 0.3
Plasma Creatinine (μmol/L)	13.7 ± 5.0	16.7 ± 1.6	12.7 ± 1.9	12.4 ± 3.0	15.1 ± 4.1	18.5 ± 4.3	8.4 ± 1.1	16.1 ± 6.1***
BUN (mmol/L)	7.9 ± 1.3	8.7 ± 1.7	10.2 ± 1.8	10.7 ± 1.6	8.5 ± 0.4	8.2 ± 1.2	11.1 ± 0.9	12.5 ± 5.3**

*Note*: Values are presented as mean ± SD.

* *p* < 0.05 compared to sham WT;

** *p* < 0.05 compared to sham KO;

*** *p* < 0.05 compared to 7dUUO WT.

### 2.3 Histology and quantification of fibrosis

For histology and immunohistochemistry, one‐half of the obstructed or sham‐operated kidneys were immersed in 4% PFA for 1 h, rinsed in PBS, dehydrated in a series of alcohol immersions in increasing concentration and embedded in paraffin. To ensure completely similar staining, the first four animals of each sex were processed further. For quantification of fibrosis, 5 μm sections were dewaxed in xylene and rehydrated again in graded ethanol. After that, the sections were stained with Picro Sirius Red and Masson's Trichrome staining to visualize collagen deposition. The tissue was imaged on an upright slide scanner (Olympus VS120) with an Olympus 20X/0.4 Plan Apochromat objective and an AVT Pike F‐505C VC50 progressive scan CCD color camera (Allied Vision Technologies). The fibrosis was quantified semiautomatic in QuPath (0.2.3) and ImageJ (NIH shareware) by thresholding to constant settings, and fibrosis was given as a percentage of total tissue area in binary images (Masson Trichrome) or red compared to total background (Sirius red).

### 2.4 Immunohistochemistry and immunofluorescence

Immunohistochemistry was performed as previously described (Therkildsen et al., 2019). Endogenous peroxidase was blocked in 35% H_2_O_2_ dissolved in methanol. Afterward, the sections were boiled in TEG buffer (1 mmol/L Tris, 0.5 mmol/L ethylene glycol tetraacetic acid, pH 9.0). For immunofluorescence, the sections were boiled in a TEG buffer for 15 min for epitope retrieval after rehydrated without blocking. Henceforth, sections were left to cool and then incubated for 30 min in 50 mM NH_4_Cl to block free aldehyde groups. Afterward, sections were incubated with blocking solution (PBS containing 1% BSA, 0.2% gelatine and 0.05% saponin) for 30 min and incubated overnight at 4°C with primary antibody (Table S1) diluted in PBS containing 0.1% BSA and 0.3% Triton X‐100. Subsequent to incubation, the sections were washed three times with PBS containing 0.1% BSA, 0.2% gelatine and 0.05% saponin. For immunoperoxidase labelling, the sections were incubated for 1 h at room temperature with horseradish peroxidase‐conjugated secondary antibody. After rinsing, the sections were incubated in 3,3′‐diaminobenzidine for 10 min to visualize the peroxidase, and counterstained in Mayer's hematoxylin. For immunofluorescence, the sections were incubated with Alexa fluor 488‐conjugated secondary antibodies diluted in PBS with 0.1% BSA and 0.3% Triton X‐100 for 30 min at RT. Sections were counterstained with 4′,6‐diamidino‐2‐phenylindole (DAPI, cat. no D1306, Molecular Probes, Fischer Scientific, Waltham, MA, USA), washed in PBS, and mounted with SlowFade Gold Antifade Mountant (cat. no S36936, Invitrogen, Waltham, MA, USA). A list of antibodies and additional information can be found in Table S1.

### 2.5 Protein isolation and immunoblotting

Total protein from mouse cortex from all mice was extracted using RIPA buffer, including phosphatase inhibitor 2 and 3 (cat. no P5726 and P0044, Sigma‐Aldrich, St. Louis, MO, USA) and a protease inhibitor cocktail tablet (cat. no 11836153001, Sigma‐Aldrich). The protein was then separated on a 12% Criterion TGX stain‐free gel (cat. no 5678125, Bio‐Rad Hercules, CA, USA), and the protein preparations were transferred to a nitrocellulose membrane (cat. no 1704159 Bio‐Rad Hercules) blocked with 5% skimmed milk in PBS‐Tween. Afterward, the membrane was washed in PBS‐Tween and incubated with specific primary antibodies (see Table S1 for target and dilution). Next, the membrane was washed with PBS‐Tween and incubated with the appropriate secondary antibody (see Table S2 for target and dilution). Finally, the membrane was incubated with the detection reagent ECL‐Prime (cat. no 12316992, GE‐healthcare, Chicago, IL, USA) and processed in the immunoblot imager (ChemiDoc MP, Bio‐Rad, CA, USA). Detected protein was normalized against total protein levels (Gürtler et al., 2013). Tissue samples from male and female mice were run in separate gels.

### 2.6 RNA isolation and QPCR

Following the manufacturer's instructions, total RNA was isolated using NucleoSpin RNAII miniKit (cat. no 740955.50, Macherey Nagel, Düren, Germany) Using spectrophotometry RNA was quantified and stored at −80°C. Synthesis of cDNA was performed by ReverseAid First Strand synthesis kit (cat. no 10387979, Thermo Scientific, Waltham, MA, USA) using 0.5 μg RNA. cDNA was subsequently used for qPCR amplification with Brilliant SYBR Green QPCR MasterMix (cat. no K0252, Thermo Scientific) following manufacturer's instructions. A table of Primer sequences is listed (Table S3).

### 2.7 Cytokines

Cytokines, TNF‐α, KC, and IL‐6 were measured using a cytometric bead array (CBA Flex Set; BD Biosciences) by flow cytometry (Accuri C6). For more details, see the manufacturer's manual (cat no 558299, 560232, 558340, 558301, and 558267, BD Cytometric Bead Array, Mouse/Rat Soluble Protein Master Buffer Kit; BD Biosciences). For the cytokine measurements, we included four additional plasma samples from P2X_7_^−/−^ female mice (sham and UUO) from the pilot study where renal tissue was not collected.

### 2.8 Quantification of extracellular staining

The amount of extracellular matrix metalloproteinase 8 (MMP8) was quantified by the following method. The fluorescent RBG pictures were divided into the respective channels (Red, blue, and Green). The red image was thresholded at a high level for automatic counting of the highly intensively stained epithelial cells in the connecting tubule and collecting duct. To detect interstitial accumulation of MMP8, the high‐intensity cells were removed by thresholding in the red image, and the green image was subtracted from the red to compensate for autofluorescence. The stained area was quantified by binarizing the green‐subtracted image (Figure S4). The threshold settings were kept constant for all images, and the quantification was carried out semi‐automated and blinded.

### 2.9 Statistical analysis

The data were analyzed in GraphPad Prism (v. 9.3.0). Data with two or more parameters were analyzed by two‐way ANOVA followed by Tukey's or Bonferroni's post hoc comparison. When data comprised of one parameter, One‐way ANOVA followed by Tukey's post hoc test or two‐tailed Students t‐test were used. Results were considered statistically significant when *p* < 0.05.

## 3 RESULTS

### 3.1 Lack of P2X_7_ receptors attenuates the development of renal fibrosis in response to UUO

**FIGURE 1:**
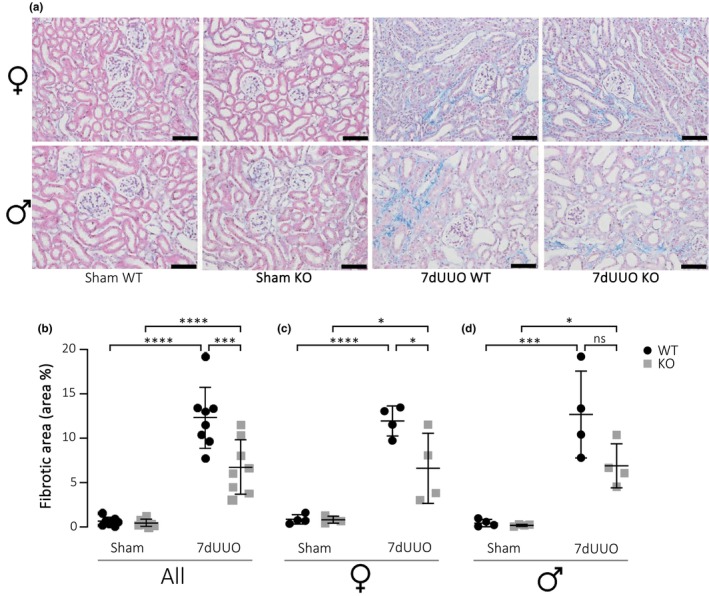
Quantification of Masson's trichrome staining in response to UUO. (a) Masson's trichrome of the renal medulla from female and male mice subjected to sham operation or 7 days UUO (7dUUO; scale bar 50 μm). (b–d) Quantification of the fibrotic areas (blue color representing collagen) in the percentage of total area from all mice (b) subjected to sham or 7dUUO, and the same data sub‐grouped into (c) female and (d) male mice. Data are presented as a scatterplot with mean ± SD (*n* = 8/4). *p < 0.05, ***p < 0.001, ****p < 0.0001.

**FIGURE 2:**
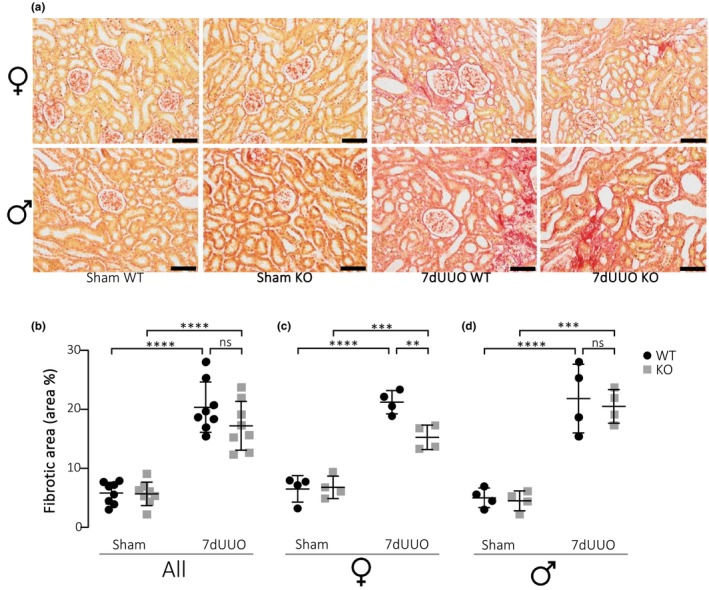
Quantification of picrosirius red staining in response to UUO. (a) Immunohistochemical staining by picrosirius red in cortical tissue from female and male mice subjected to sham operation or 7 days UUO (scale bar 50 μm). (b–d) Quantification of picrosirius red staining of the renal cortex from all mice (b) subjected to sham or 7dUUO, and the same data sub‐grouped into (c) female and (d) male mice. The bright red color represents collagen fibers. Each bar represents mean ± SD (*n* = 6). **p < 0.01, ***p < 0.001. Data are presented as a scatterplot with mean ± SD (*n* = 8/4). **p < 0.01, ***p < 0.001, ****p < 0.0001.

Previous data have shown that the P2X_7_ receptor is central in the development of tissue fibrosis (Bhakdi & Tranum‐Jensen, 1988; Brown et al., 2013; Huang et al., 2014; Riteau et al., 2010; Therkildsen et al., 2019). We have recently demonstrated that inhibition or lack of P2X_7_R significantly dampens renal fibrosis in response to pyelonephritis induced with uropathogenic *E. coli* in female mice (Therkildsen et al., 2019). The fibrosis in response to pyelonephritis is discrete and primarily affects the cortical regions in focused areas along the proximal tubules. This study was carried out in female mice because urinary tract infections are primarily a female‐associated condition. There have been deviating findings regarding the P2X_7_ receptor in experimental models for more severe renal fibrosis (Goncalves et al., 2006; Prendecki et al., 2022; Therkildsen et al., 2019). Here, we use unilateral ureteral obstruction (UUO) to evaluate the effect of lacking the P2X_7_ receptor and review the potential effect of gender on the overall response. The level of fibrosis was determined by two staining methods: trichrome (Figure 1) and Sirius Red (Figure 2). Mice exposed to 7dUUO displayed substantial fibrosis (Figure 1a), and this response appeared similar in female and male mice, although the response was much more variable in males. The fibrotic area was reduced in the UUO mice lacking P2X_7_R from 12.3% fibrosis in wildtype to 6.79% in P2X_7_^−/−^ mice (*p* < 0.0008). This effect was most pronounced in the female mice, where the fibrotic area was reduced by 55%. The increase in fibrosis upon UUO was confirmed by Sirius Red stain (Figure 2). However, when evaluating the fibrosis by this method, the reduction in fibrosis in female P2X_7_^−/−^ mice was more modest. Moreover, we did not detect a statistically significant reduction in fibrotic areas in males alone, although there was a tendency toward reduction. Collectively, lack of P2X_7_^−/−^ has a mild antifibrotic effect in response to UUO in female mice.

### 3.2 Basic characteristics of plasma parameters

The body weight was similar in all groups of female mice. However, the male P2X_7_^−/−^ mice were statistically significantly larger than the wildtype, and the P2X_7_^−/−^ males experienced a drop in body weight in the 7‐day post operation observation period (Table 1). In terms of renal function, the female mice exposed to UUO showed larger kidneys than the sham controls both in the P2X_7_^+/+^ and P2X_7_^−/−^. However, in the males, UUO‐induced increase in kidney size was only observed in wildtypes (Table 1). Overall data on renal function was similar in the P2X_7_^+/+^ and P2X_7_^−/−^ mice. The only surprising finding was that female P2X_7_^−/−^ showed reduced plasma K^+^ concentration, whereas the plasma Na^+^ concentration was slightly higher than the 7dUUO wildtype mice. This finding suggests a degree of renal potassium wasting via the remaining functional kidney in these mice.

### 3.3 P2X_7_R expression did not affect myofibroblast accumulation

**FIGURE 3:**
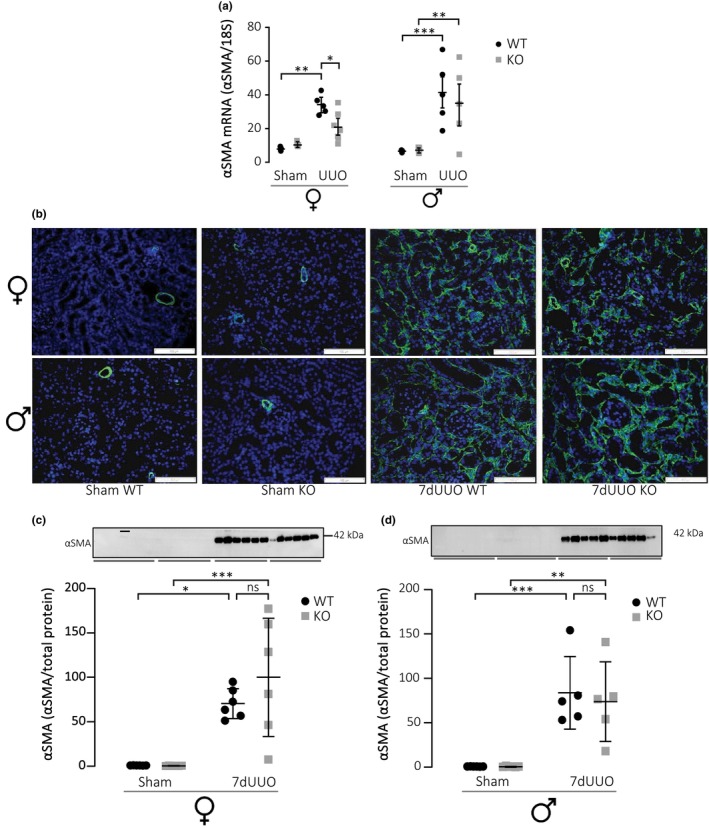
Quantification of changes in α‐SMA in response to UUO. (a) Represent mRNA level of α‐SMA relative to 18S for female and male mice. (b)Immunofluorescence staining of α‐SMA in cortex from female and male mice subjected to UUO or sham operation. The green fluorescence represents α‐SMA and blue color (DAPI) stain nuclei (scale bar 100 μm). (c, d) Represent protein expression of α‐SMA for female (c) and male mice (d), analyzed as protein band intensity relative to total protein (Gürtler et al., 2013). Data are presented as scatterplots including mean ± SD (*n* = 8/4). *p < 0.05, **p < 0.01, ***p < 0.001.

Our previous data support the notion that P2X_7_ activation is not responsible for the initial renal damage per se but is activated during repair (Therkildsen et al., 2019). Consistent with the P2X_7_Rs being primarily expressed in bone‐marrow‐derived cells (for review, see De Marchi et al., 2021), the P2X_7_R has implications for immune cell activation that leads to lay down of additional extracellular matrix (Bhakdi & Tranum‐Jensen, 1988; Brown et al., 2013; Huang et al., 2014; Riteau et al., 2010). In the pyelonephritis model, the discrete, localized fibrosis did not result in a measurable increase in epithelial‐myofibroblast transformation when measured by immunoblotting of the entire cortex. This type of transformation is more easily detected in a model of extensive cell damage, as is the case in fibrosis in response to UUO (for review, see Wyczanska & Lange‐Sperandio, 2020). Accordingly, UUO markedly increased α‐smooth muscle actin (α‐sma) mRNA abundance in both females and males, consistent with more cells having converted into a more myofibroblast‐like phenotype (Figure 3a). This response was markedly blunted in the P2X_7_R in female knockout mice, whereas no difference was detected in the males. The immunofluorescence‐stained renal sections confirmed a rise in α‐SMA expression in response to UUO, without any appeared discrepancies between the genotypes (Figure 3b). This was, however, not the case for the males where the pattern was similar regardless of the genotype (Figure 3b). This was confirmed by immunoblotting for total α‐SMA, which increased to similar levels regardless of the P2X_7_ expression (Figure 3c, d).

### 3.4 P2X_7_ ^−/−^ mice show less matrix accumulation in response to UUO

**FIGURE 4:**
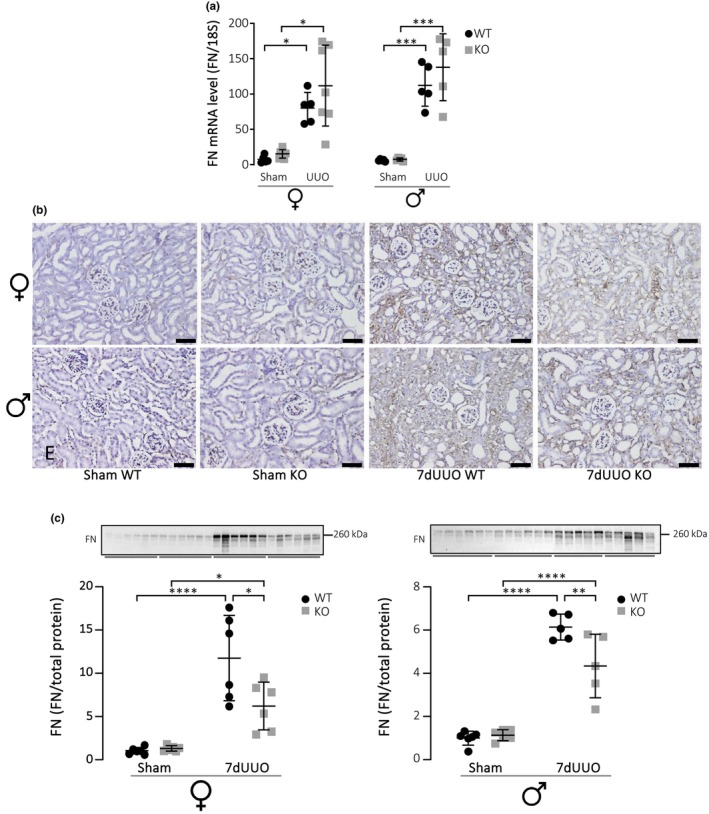
Quantification of changes in fibronectin in response to UUO. (a) Shows mRNA level of fibronectin (FN) relative to 18S. (b)Immunohistochemical labelling of fibronectin (FN) in cortex from female and male mice subjected to UUO or sham (scale bar 50 μm). (c)Representative immunoblots of fibronectin for female and male mice analyzed as protein band intensity relative to total protein (Gürtler et al., 2013). Data are presented as mean ± SD (*n* = 6). *p < 0.05, ***p < 0.001.

**FIGURE 5:**
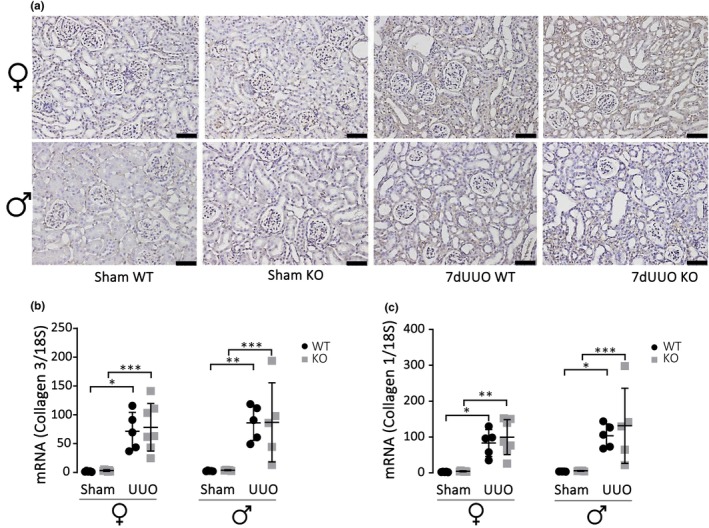
Changes in collagen‐1 and collagen‐3 in response to UUO. (A) Immunohistochemical staining of collagen‐3 in the cortex from female and male mice subjected to UUO or sham (scale bar 50 μm). (b) Corresponding levels of mRNA for collagen‐3 relative to 18S for mice. (c) mRNA level of collagen‐1 relative to 18S mice subjected to UUO or sham. Data are presented as a scatterplot with mean ± SD (*n* = 6). *p < 0.05, ***p < 0.001.

Consistent with an increased the abundance of myofibroblasts, UUO is known to markedly increases abundance of the key matrix proteins tissue fibronectin, collagen 1 and collagen 3 (Ahmad et al., 2022). We confirmed that fibronectin, which is a prerequisite for the fibrotic process, was still increased in the tissue 7 days after UUO (Figure 4) and this increase was observed in both males and females. This response was markedly attenuated in the female P2X_7_^−/−^ mice, appreciated both in the immunohistochemistry data (Figure 4b) and in the immunoblots (Figure 4c), where the fibronectin content in the cortex was approximately 50% lower in female P2X_7_^−/−^ compared to wildtype (*p* = 0.0143). This effect was again less pronounced in males, where the expression of fibronectin was slightly but statistically significantly reduced to about 30% in P2X_7_^−/−^ mice compared to P2X_7_^+/+^ controls (Figure 4c, *p* = 0.0093). Interestingly, the increased transcription of fibronectin did not result from increased mRNA levels for fibronectin (Figure 4a). Clearly, the mRNA levels for fibronectin were increased in response to 7dUUO; however, if anything, the mRNA levels seem to be a little elevated in the P2X_7_^−/−^ mice. This could potentially suggest that P2X_7_‐dependence occurs on the transcription level or that the fibronectin response is delayed by P2X_7_R deficiency. Correspondingly, we found increased expression levels of collagen 3 in response to 7dUUO, both in males and females. The immunohistochemistry suggests the amount of tissue collagen 3 to be reduced in both males and females the P2X_7_^−/−^ compared to control (Figure 5a). This apparent difference is not based on a regulation of mRNA level since the mRNA levels are increased to similar levels both for collagen 3 (Figure 5b) and collagen 1 (Figure 5c). Collectively, these finding may suggest that the lack of P2X_7_R does prevent initiation of the fibrotic process but is a determinator of the final amount of extracellular matrix laid down in response to a profibrotic activator.

### 3.5 Neutrophil–macrophage balance

**FIGURE 6:**
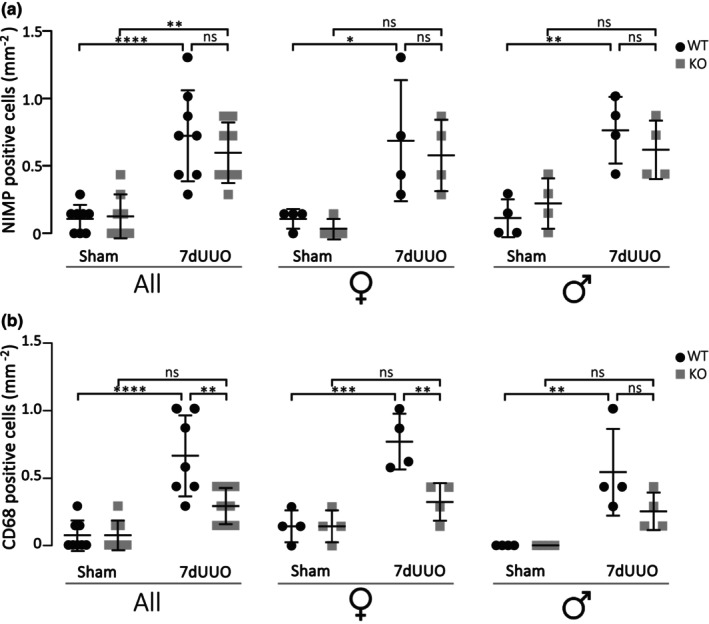
Changes in neutrophils and macrophages in response to UUO. (a) Number of neutrophil granulocytes per centimeter square counted as anti‐NIMP‐R14‐positive cells in renal cortex in total, and the same data sub‐grouped into male and female mice subjected to UUO or sham. (b) Number of macrophages counted as anti‐CD68 stained cells in renal cortex from female mice subjected to UUO or sham. The quantification was conducted blinded and presented as mean ± SD. *p < 0,05, **p < 0.01, ***p < 0.001, ****p < 0.0001; *n* = 4–8.

We have previously shown that the acute neutrophil response tends to linger in the renal cortex in P2X_7_^−/−^ mice with a corresponding delayed or reduced macrophage infiltration (Therkildsen et al., 2019). Thus, we tested whether this was also the case in a 7dUUO model. Figure 6 shows the number of neutrophil granulocytes (NIMP‐positive cells, Figure 6a) and macrophages (CD‐68‐positive cells, Figure 6b) in the renal cortex. Seven days of UUO markedly increased the number of neutrophils around six‐fold from 0.108 ± 0.036 NIMP positive cells per mm^2^ to 0.725 ± 0.119 NIMP positive cells per mm^2^ (*p* < 0.0001, *n* = 8), and this increase was similar in P2X_7_^+/+^ and P2X_7_^−/−^, and there was no statistically significant difference among female and males (Figure 6a). Similarly, 7dUUO resulted in increased infiltration of macrophage, with a 13‐fold increase in CD68 positive cells from 0.048 ± 0.031 to 0.628 ± 0.013 CD68 positive cells per mm^2^ (*p* < 0.0001). Interestingly, the UUO‐induced renal macrophage infiltration was markedly blunted in P2X_7_^−/−^ mice with 0.290 ± 0.047 CD68 positive cells per mm^2^ (*p* = 0.0018). Dividing the data into gender, the female P2X_7_^−/−^ mice had statistically significantly less macrophage infiltration compared to WT, whereas this was only a trend in the males.

The notion that the P2X_7_R is working downstream in the fibrotic process was supported by data on the TGFβ mRNA levels in the renal tissue. We were unable to detect any indication of altered TGFβ signaling between P2X_7_^−/−^ and P2X_7_^+/+^ mice, mRNA levels were similar for TGFβ in the renal cortex (Figure S2). However, the P2X_7_R is, as mentioned, implicated in inflammasome activation and thus, in the release of the early proinflammatory cytokines. However, in the 7d‐UUO model, the inflammatory response to ureteral obstruction has peaked, and thus, it was not surprising to find that the relatively low levels of proinflammatory cytokines in the plasma of the mice, whereas the keratinocyte chemoattractant (KC) and interleukin‐6 (IL‐6) was still statistically significantly elevated in female UUO mice. The response to UUO was similar in both genotypes. (Figure S3).

**FIGURE 7:**
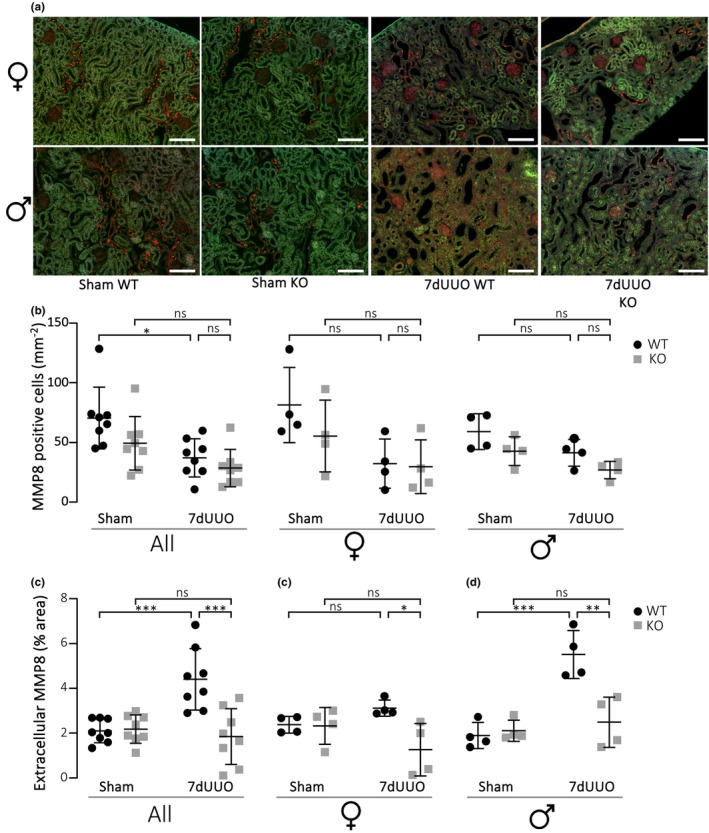
Metalloproteinase 8 (MMP8) in renal extracellular matrix. (a) Immunofluorescence staining of MMP‐8 (red) in cortical tissue from female and male mice (scale bar 100 μm). (b) Number of MMP‐8‐positive cells in the cortex per area, counted by an automated classifier based on intensity, size and form, from mice in total and separately in female and male mice subjected to UUO or sham. The quantification was conducted blinded and presented as a scatterplot with mean ± SD (*n* = 8/4), *p < 0.05 (c) shows a quantification of extracellular MMP8 (red extracellular stain) in the percentage of total area of renal cortex from mice subjected to either sham or UUO. The quantification was conducted blinded and by automated scrips subtracting autofluorescence (green) background from red image and thresholding. Data are presented as a scatterplot with mean ± SD (*n* = 8/4), *p < 0.05, ***p < 0.001.

Our data suggest an association between reduced matrix accumulation and reduced macrophage infiltration in the P2X_7_^−/−^ mice. The question remains: why does the lack of P2X_7_Rs reduce macrophage recruitment? Interestingly, we found that metalloprotease 8 (MMP8) released to the tissue is markedly reduced in the P2X_7_^−/−^ mice compared to controls (Figure 7). Extracellular MMP8 is known to be involved in instigating macrophage migration (Wu et al., 2021). Our data show that the amount of MMP8 in the interstitium increases dramatically in response to 7dUUO in the wildtype (Figure 7a,c), whereas interstitial MMP8 stays at baseline levels in P2X_7_^−/−^ mice exposed to UUO. The increase in extracellular MMP8 in response to UUO is mainly seen in males. However, the MMP8 level in the interstitium is nevertheless depressed in both male and female P2X_7_^−/−^ mice compared to wildtype (male *p* = 0.0028, female *p* = 0,0417). MMP8 is most likely released to the interstitium from recruited neutrophils. However, we did observe that MMP8 was distinctly expressed in the apical domain in a subpopulation of epithelial cells of the collecting duct. Based on morphology, these are likely to be intercalated cells. The tissue distribution of MMP seemed to be specific to MMP8 since MMP9 steadily marked primarily invading immune cells, with a pattern that fits macrophage infiltration, and with a marked higher number of MMP9 positive cells in wildtype‐mice exposed to 7dUUO compared to P2X_7_^−/−^ mice (Figure S1). Taken together, our data show that reduced P2X_7_R function dampens the amount of matrix accumulation during renal fibrosis, primarily by reducing the macrophage response.

## 4 DISCUSSION

Renal fibrosis is strongly associated with the progression of CKD, irrespective of the underlying pathogenesis. At present, it is not settled whether the process of tissue fibrosis is causatively associated with the progression of renal insufficiency or merely a by‐product of the ongoing tissue damage that reduces renal function. Should the fibrotic process as a separate entity in itself not cause extra nephron loss, the excess extracellular matrix will disturb the delicate diffusion processes necessary for optimal renal function. Thus, it would be rational to dampen matrix accumulation in patients suffering from CKD without interfering with tissue healing in response to renal damage.

In a model of renal fibrosis secondary to *E. coli*‐induced pyelonephritis, we were able to show that P2X_7_R‐deficient mice were protected against renal fibrosis without interfering with the earlier stage of renal damage, inflammation and activation of tissue fibroblasts (Therkildsen et al., 2019). In support of the notion that P2X_7_Rs are not involved in early renal damage, it has been demonstrated that the lack of P2X_7_Rs did not influence the renal damage seen in models of glomerulonephritis and autoimmune vasculitis, including the activation of fibroblasts measured as increased α‐SMA expression (Prendecki et al., 2022). Moreover, various reports about the P2X_7_R in renal damage and fibrosis have been inconsistent (Goncalves et al., 2006; Luz et al., 2019; Xu et al., 2022), questioning the P2X_7_ as a relevant target for interference against fibrosis. The inconsistency has largely been ascribed to challenges with the models for studying the P2X_7_R function. The P2X_7_R‐deficient mouse used in this study was originally developed by GSK and has been proven not to be a complete knockout (Masin et al., 2011). The ideal knockout mouse for P2X_7_ is not available, in as much as the knockout mouse developed by Pfizer also has residual P2X_7_R function (Nicke et al., 2009). In addition to the mice models, a P2X_7_ receptor‐deficient rat is also available. However, the P2X_7_R‐deficient rat has a limited phenotype and has residual effects of P2X_7_ specific antagonists (Prendecki et al., 2022). It must be noted that the GSK P2X_7_^−/−^ mouse on BALB/cJ background used here has a distinct phenotype in terms of renal fibrosis, which has previously been found to be comparable to P2X_7_ receptor inhibition (Therkildsen et al., 2019). Moreover, we have confirmed that the P2X_7_ receptor function is undetectable in peritoneal macrophages from P2X_7_^−/−^‐BALB/cJ (Fagerberg et al., 2016). Despite this, we cannot exclude that we might underestimate the true effect of the P2X_7_R on UUO‐induced renal fibrosis by using GSK P2X_7_^−/−^ model.

With these reservations, we consistently find that the amount of matrix protein is lower in the P2X_7_^−/−^ compared to the wildtype in response to 7 days of UUO. This effect was not statistically significant when the males were analyzed as a subgroup, most likely a result of the low number of observations for the subgroup analysis. The quantification of the overall fibrosis is paralleled by the amount of fibronectin and the amount of collagen 3. This is observed even though the cellular production of the matrix protein apparently continues undisturbed by the expression of the receptor since the corresponding mRNA levels are, if anything, slightly higher for fibronectin and collagen 3 in tissue isolated from the P2X_7_^−/−^ mice. This discrepancy could potentially suggest that the fibrotic process is delayed in the P2X^−/−^ mice and that the mice are caught in a time window where the mRNA levels are high, but this has not yet manifested as a marked increase in matrix proteins compared to the wildtype. Another option is that the balance between the accumulation and degradation of matrix protein is altered in the P2X_7_^−/−^ mice. Our previous study shows that the accumulation of macrophages in response to inflammation is very much dampened in the renal cortex of P2X_7_^−/−^ mice compared to controls (Therkildsen et al., 2019). Reversely, the P2X_7_^−/−^ mice retain their neutrophils and continue to have high numbers of neutrophils in the cortex long after they have cleared in the control mice (Therkildsen et al., 2019).

The reduced recruitment of macrophages in the P2X_7_ deficient mice was confirmed in both male and female mice. Since macrophages are responsible for a large degree of stimulation of tissue fibrosis (for review, see Binatti et al., 2022; Ortiz‐Zapater et al., 2022; Park et al., 2022), the reduced fibrosis in the P2X_7_^−/−^ is easily explained by the reduced number of recruited macrophages to the renal tissue. Moreover, macrophages are responsible for removing senescent or damaged neutrophils (Savill et al., 1989) during inflammation. Through that mechanism, macrophages lessen the potential release of tissue‐damaging proteases, etc. from activated or damaged neutrophils. Therefore, a reduced number of macrophages may tip the balance between neutrophil‐dependent matrix degradation and macrophage‐stimulated matrix generation, overall dampening the build‐up of extracellular matrix. Recent data confirm our notion that the balance between neutrophils and macrophages is dictating the degree of fibrosis after an *E. coli* infection (Ruiz‐Rosado et al., 2021). This study suggests fibrosis dampened via macrophage‐specific inhibitory antibodies (Ruiz‐Rosado et al., 2021). However, our data propose that one can obtain an equally beneficial effect by targeting P2X_7_Rs. Even though macrophages are directly involved in the development of fibrosis, which correlates to a negative outcome of renal diseases (Eardley et al., 2008; Yoshimoto et al., 2004), their role in fibrosis is more complex. Macrophages has been subdivided into M1 (classical, pro‐inflammatory) and M2 (alternatively activated, anti‐inflammatory macrophage) types based on their in vitro differentiation during specific cytokine exposure. The role of M2 macrophages is widely discussed, as they appear to play both an anti‐fibrotic and fibrotic role in the kidney, depending on disease type and stage (for review, see Braga et al., 2015). In this study, we did not subdivide the macrophages recruited to the kidney because the macrophage subtype pattern in vivo is more fluid (Strizova et al., 2023), and an M1/M2 division in a cross‐sectional study like ours would not do the topic justice. However, one could speculate that a reduced fraction of macrophages found in the female P2X_7_ knockout renal cortex are of M2‐like subdivision. The argument is that the female mice seemingly have lower levels of TNF‐α and a significantly higher KC response, which both would favor M2‐like polarization (Xiao et al., 2018; Yao et al., 2019). Regardless of the relative contribution of subpopulations, the total number of macrophages was reduced in P2X_7_ knockout mice, which is likely to play an elementary role for the phenotype.

Our data do not immediately explain why macrophage infiltration is reduced in the P2X_7_‐deficient mice compared to the wildtype. The P2X_7_ receptor is expressed in a variety of bone marrow‐derived and tissue‐resident immune cells as several subtypes of T‐lymphocytes, mast cells, monocytes, and neutrophils (for review, see Di Virgilio et al., 2017; Pelegrin, 2021). However, macrophages and monocytes are known as one of the prime expressors of P2X_7_R, implicated in the NLRP3 inflammasome and IL‐1β processing (Franceschini et al., 2015; Kahlenberg & Dubyak, 2004; Lister et al., 2007; Pelegrin et al., 2008; Perregaux & Gabel, 1994). However, although purinergic signaling promotes macrophage migration, this effect is very unlikely to be mediated via the P2X_7_ receptor (Kronlage et al., 2010), and thus, it is not reasonable to assume that the reduced number of macrophages in the renal cortex is caused by a reduced ability of the macrophages to migrate. Activated neutrophils release a variety of cytokines and enzymes. Of those, metalloprotease 8 or neutrophil collagenase is known to be stored in distinct neutrophil granules (Murphy et al., 1977; Rindler‐Ludwig & Braunsteiner, 1975) that are released in response to activation (Ohlsson & Olsson, 1977). Released MMP8 has been shown to be involved in macrophage migration toward damage/inflammation site (Wu et al., 2021), and consistently MMP8 deficient mice are protected against bleomycin‐induced lung fibrosis (Garcia‐Prieto et al., 2010). Reversely, it has recently been shown that tissue inhibitor of metalloproteinase 1 (TIMP1) expression, which inhibits the activity of most of the metalloproteases, including MMP8 (for review, see Visse & Nagase, 2003), dampens tissue fibrosis (Kokeny et al., 2022). Moreover, the authors specifically demonstrate that inhibition with anti‐TIMP‐1 markedly increased tissue fibrosis in mice (Kokeny et al., 2022). In this light, our data suggest that the P2X_7_ promotes macrophage migration by increasing the amount of MMP8 present in the interstitium and preventing this dampening the overall accumulation of matrix components in response to renal injury.

The question is which cell type is responsible for MMP8, which is clearly released to the renal interstitium during fibrosis. As mentioned, MMP8 has notoriously been shown to be expressed by neutrophils (Murphy et al., 1977; Rindler‐Ludwig & Braunsteiner, 1975), and neutrophils do express functional P2X_7_R that have been shown to be involved in inflammasome activation and IL‐1β processing (Karmakar et al., 2016). P2X_7_R activation has been shown to regulate MMP9 expression indirectly via IL‐1β signaling in atherosclerotic vessels (Lombardi et al., 2017), but there is no apparent connection between P2X_7_ and MMP8. However, P2X_7_ activation has been demonstrated to increase the intracellular Ca^2+^ concentration in neutrophils, and neutrophils are known to store MMP8 in tertiary granules release by degranulation (for review, see Zhang et al., 2022). Thus, P2X_7_ activation is likely to increase the release of MMP8, and the observed increase in interstitial MMP8 could originate from activation from invading neutrophils. This notion would fit the reduced tissue expression of MMP8 observed in the P2X_7_^−/−^ mouse. However, we made the peculiar observation that sporadic cells in the collecting duct, which, based on morphology, are likely to be intercalated cells, express MMP8 in their apical domain. MMP8 has previously been suggested to be expressed in the kidney without being associated with any specific segment or cell type (for review, see Zakiyanov et al., 2019). Therefore, we cannot exclude the possibility that MMP8 is released from the epithelial cells.

Consistently, we find that female mice have a more pronounced reduction in fibrosis in the absence of P2X_7_^−/−^. This supports previous findings that the female sex protects against renal fibrosis, an effect that has been shown mainly to be a result of decreased testosterone levels rather than an estrogen effect (Hewitson et al., 2012, 2016). We do not find a higher degree of renal fibrosis in males compared to females in this study. However, with up to 20% of the renal tissue being fibrotic within 7 days, we may approach a ceiling effect where a difference is hard to detect. The previously reported relative protection against fibrosis by the female sex is uncovered in the P2X_7_^−/−^ mice, which is clearly less profibrotic. These findings can potentially also explain some of the variability in the literature regarding a fibrotic phenotype of P2X_7_ receptor‐deficient mice and underline the importance of including gendered data and considering models with milder perturbations to induce fibrosis.

In conclusion, the lack of P2X_7_Rs dampens the fibrotic response to UUO by reducing macrophage infiltration secondary to renal injury and the resulting acute inflammation. We suggest that the P2X_7_ receptors effect is most pronounced in the signaling interphase between neutrophils and macrophages in an MMP8‐dependent fashion. Based on our results, we propose that P2X_7_ receptors may be exploited as a target for the long‐term prevention of tissue fibrosis, slightly diminishing the overall matrix accumulation without seriously affecting tissue inflammation and healing.

## FUNDING INFORMATION

Grant support was provided by Independent Research Fund Denmark|Medical Sciences (Danmarks Frie Forskningsfond|sundhed og sygdom, 0602‐02145B and 1331‐00203A).

## CONFLICT OF INTEREST STATEMENT

None of the authors have any conflicts of interest to declare.

## Supporting information


Figure S1:

Table S1.

## Data Availability

The data that support the findings of this study are available on request from the corresponding author. The data are not publicly available due to privacy or ethical restrictions.
