# Supplementary material for: P2X7 accelerate tissue fibrosis via metalloproteinase 8‐dependent macrophage infiltration in a murine model of unilateral ureteral obstruction
Source: Physiol Rep. 2023 Nov 23;11(22):e15878. doi: 10.14814/phy2.15878 (PMC10665779; doi:10.14814/phy2.15878)

Supplementary figure 1

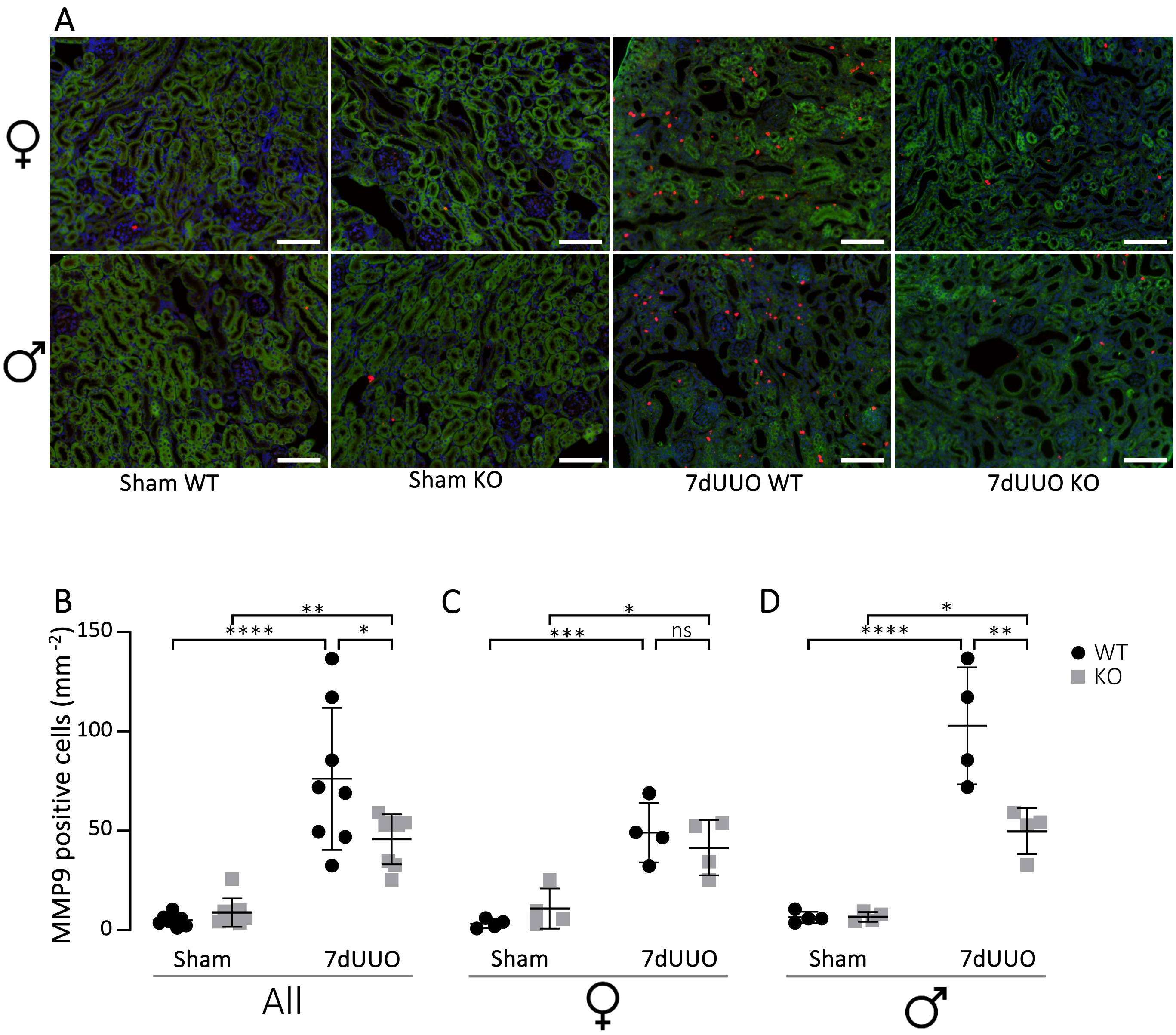

Supplementary figure 1:Metalloproteinase 9 (MMP9) positive cells in renal cortex. (A) *shows immunofluorescence staining of MMP-9 (red) in cortical tissue from female* ***(A-D)*** *and* ***(E-H)*** *male mice (scale bar 100 µm).* ***(I-K)*** *shows the number of MMP-9 positive cells in the cortex per area, counted by an automated classifier based on intensity, size and form, from mice in total* ***(I)****, female* ***(J)*** *and male* ***(K)*** *mice subjected to UUO or sham. The quantification was conducted blinded and presented as a scatter plot with mean ± SD (n=8/4), * p<0.05, ** p<0.01, *** p<0.001 and **** p<0.0001.*

Supplementary figure 2

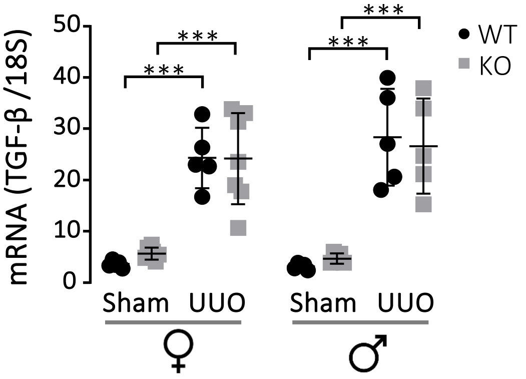

Supplementary figure 2: Changes in TGF-β mRNA in response to UUO

*The figure illustrates mRNA level of TGF-β relative to 18S for female and male mice exposed to sham or 7dUUO. Data are given as mean±SD (n=6) and p< 0.001 is signified by ***.*

Supplementary figure 3

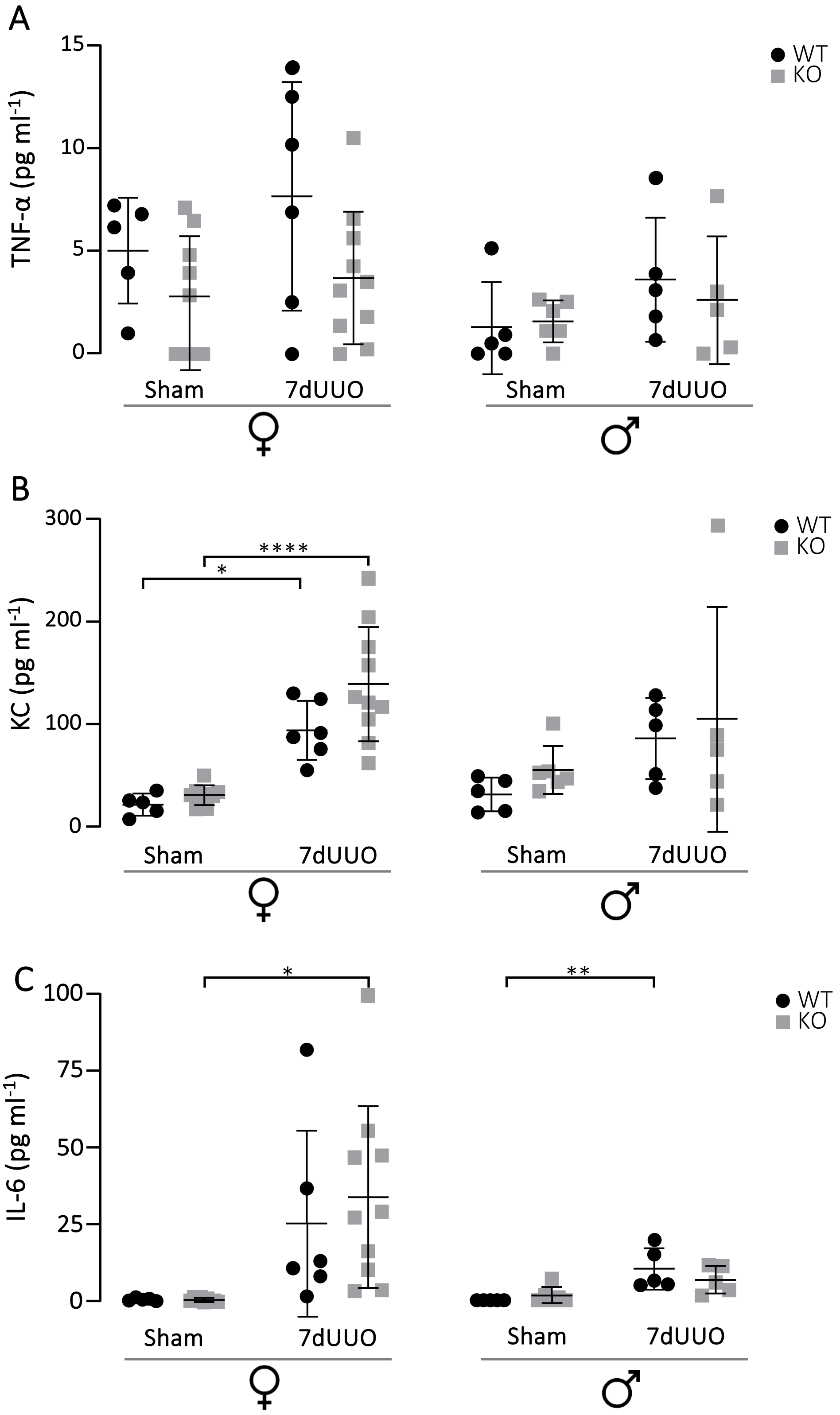

Supplementary figure 3: Pro-inflammatory cytokines in urine after 7 days of UUO

The figure illustrated the concentration of (**A**) TNF-α, (**B**) keratinocyte chemoattractant (KC), and (**C**) interleukin-6 (IL-6) in the urine of female and male mice subjected to sham or seven days of UUO. The data are shown as mean±SD (n=5-8).

Supplementary figure 4

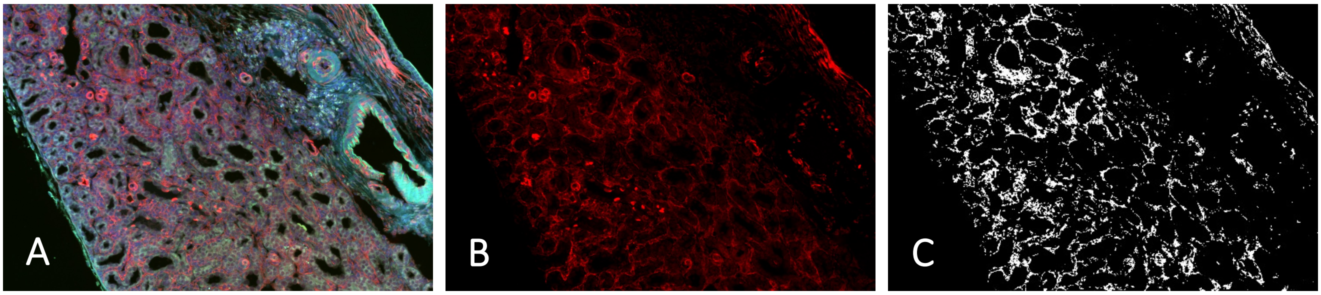

Supplement: Supplementary file 1 — Figure S1: [file PHY2-11-e15878-s001.pdf]
