# Supplementary material for: P2X7 accelerate tissue fibrosis via metalloproteinase 8‐dependent macrophage infiltration in a murine model of unilateral ureteral obstruction
Source: Physiol Rep. 2023 Nov 23;11(22):e15878. doi: 10.14814/phy2.15878 (PMC10665779; doi:10.14814/phy2.15878)
Supplement: Supplementary file 2 — Table S1. [file PHY2-11-e15878-s002.pdf]

Supplementary table 1

Primary antibodies

Target	Catalog no.	Company	Species	Dilution
**αSMA**	M0851	Dako	Mouse	1:2000
**FN**	ab2413	Abcam	Rabbit	1:50
**COL3A1**	ab7778	Abcam	Rabbit	1:10
**NIMP-R14**	ab2557	Abcam	Rat	1:500
**CD68**	ab125212	Abcam	Rabbit	1:900
**MMP8**	ab53017	Abcam	Rabbit	1:1500
**MMP9**	ab38898	Abcam	Rabbit	1:1000

Supplementary table 2

Secondary antibodies

Target	Catalog no.	Company	Species	Dilution
IF	A11001	Life Technologies	Goat anti-mouse	1:400
IF	A11036	Life Technologies	Goat anti-rabbit	1:400
WB, IHC	P0448	DAKO	Goat anti-rabbit	1:2000
WB, IHC	P0447	DAKO	Goat anti-mouse	1:2000
IHC	6180-05	Southern Biotech	Rabbit anti-rat	1:100

Supplementary table 3

Primer sequences

Target gene	Accession no.	Forward	Reverse
αSMA	NM_007392.3	5´-CTGACAGAGGCACCACTGAA-3´	5´-CATCTCCAGAGTCCAGCACA-3´
FN	NM_010233.2	5´-AATGGAAAAGGGGAATGGAC-3´	5´-CTCGGTTGTCCTTCTTGCTC-3´
TGF-β	NM_011577.2	5´-ACCGGAGAGCCCTGGATAC-3´	5´-TTCTCTGTGGAGCTGAAGCA-3´
COL1A1	NM_007742.4	5´-CACCCTCAAGAGCCTGAGTC-3´	5´-ACTCTCCGCTCTTCCAGTCA-3´
COL3A1	NM_009930.2	5´-GCACAGCAGTCCAACGTAGA-3´	5´-TCTCCAAATGGGATCTCTGG-3´
18S	NM_011296.2	5´-GAAAATAGCCTTCGCCATCA-3´	5´-TCCCATCCTTCACATCCTTC-3´
